# Incidence of Bloodstream Infections in Pediatric Patients with Cancer during Febrile Neutropenia: A Retrospective Study

**DOI:** 10.31662/jmaj.2024-0369

**Published:** 2025-03-28

**Authors:** Yukihiro Matsukawa, Jun Matsubayashi, Kenichi Sakamoto, Kohei Takashima, Yuhachi Ikeda, Makoto Osawa, Takashi Taga, Yoshihiro Maruo

**Affiliations:** 1 Department of Pediatrics, Shiga University of Medical Science, Otsu, Japan; 2 Center for Clinical Research and Advanced Medicine, Shiga University of Medical Science Hospital, Otsu, Japan; 3 Division of Infection Control and Prevention, Shiga University of Medical Science Hospital, Otsu, Japan

**Keywords:** acute myeloid leukemia, bloodstream infections, febrile neutropenia, high-dose cytarabine, pediatric cancer

## Abstract

**Introduction:**

Bloodstream infections (BSIs) are a major concern in pediatric patients with cancer, especially during episodes of febrile neutropenia (FN). In this study, we aimed to evaluate the incidence of BSI across various pediatric malignancies and identify cancer subtypes associated with a heightened risk of BSI.

**Methods:**

This single-center, retrospective cohort study analyzed the electronic medical records of pediatric patients with cancer treated between April 2012 and March 2023. Eligible patients included those diagnosed with acute myeloid leukemia (AML), acute lymphoblastic leukemia (ALL), lymphoma, brain tumors, and solid tumors. For each chemotherapy course, we assessed BSI incidence and FN episodes.

**Results:**

This study included 152 pediatric patients who underwent 829 chemotherapy courses. The cohort comprised 21 patients with AML, 52 with ALL, 10 with lymphoma, 26 with brain tumors, and 43 with solid tumors. Compared to other cancer types, the AML group exhibited the highest proportion of BSI across all chemotherapy courses (17% vs. 4%-7%). During FN episodes, the AML cohort had a significantly higher BSI incidence (22%) than other groups (7%-13%). Notably, chemotherapy courses involving high-dose cytarabine (HD-CA) had a substantially higher BSI incidence (30%) among the patients with AML than courses without HD-CA (2%).

**Conclusions:**

Patients with AML have the highest risk of BSIs in pediatric malignancies, particularly during FN episodes. In addition, our findings highlight an association between BSIs and HD-CA use in patients with AML.

## Introduction

Febrile neutropenia (FN) is the most common complication encountered during pediatric cancer therapy ^(1)^. Bloodstream infections (BSIs) are the most severe manifestation of FN, affecting 10-30% of pediatric patients with cancer during FN episodes ^(2)^. However, no consensus has been established regarding the optimal management of fever without neutropenia, with BSIs reported in 4.7% of such cases ^(3)^.

BSIs are a major contributor to mortality in pediatric patients with cancer ^(4), (5)^. Regardless of the neutropenic status, the incidence of BSIs varies per patient according to the cancer type, occurring in 27% of patients with acute lymphoblastic leukemia (ALL), 48% with acute myeloid leukemia (AML), 10% with lymphoma, 12% with solid tumors, and 14% with brain tumors ^(6)^. In adult studies, the incidence of BSIs during FN episodes reportedly ranges from 11%-38% ^(7)^, with mortality rates significantly higher in patients with neutropenia than in those without ^(7)^. Consequently, BSIs associated with FN require greater clinical attention than those occurring without neutropenia.

In this study, we aimed to evaluate the incidence of BSIs across various pediatric cancer types, specifically focusing on BSI occurrence during FN episodes, and identify the cancer types associated with a heightened risk of BSIs.

## Materials and Methods

### Patient selection

This retrospective study included newly diagnosed pediatric patients (<18 years old) with cancer who received chemotherapy during hospitalization at Shiga University of Medical Science Hospital between April 2012 and March 2023. The eligible patients were those diagnosed with AML, ALL, lymphoma, brain tumors, or solid tumors, classified according to the International Classification of Childhood Cancer, 3rd edition ^(8)^. We collected data until autologous or allogeneic hematopoietic stem cell transplantation was initiated.

This study was conducted in accordance with the principles outlined in the Declaration of Helsinki and was approved by the Institutional Ethics Committee of Shiga University of Medical Science (Approval Number R2023-074). The requirement for informed consent was waived due to the study’s retrospective nature. However, a summary of the study protocol was made publicly available on the institution’s website, allowing patients to opt out if they desired.

### Data collection

We retrospectively extracted patients’ clinical data from electronic medical records, including variables such as age, sex, presence of intravenous catheters, cancer diagnosis, treatment regimen, chemotherapy details, number of treatment courses, incidence of FN episodes per treatment course, FN occurrence dates, BSI occurrence dates and outcomes, isolated blood culture pathogens, and treatment-related mortality. The initiation date of each treatment course was considered to be day 1. We also recorded the duration of the period when the neutrophil count was below 500/μL or 200/μL for the chemotherapy courses administered between January 2020 and March 2023.

### Definitions of FN and BSI episodes

FN was defined as an episode of fever (a single recorded axillary temperature >38.0°C) occurring during neutropenia (absolute neutrophil count <0.5 × 10^9^/L). BSI was defined as the presence of a positive blood culture result. Blood cultures were considered clinically significant if a pathogen was isolated within 48 h, as contaminants are typically identified after 48-72 hours ^(9)^.

### Statistical analysis

We calculated the FN incidence for each cancer type and determined the proportion of BSIs based on the total number of treatment courses and FN episodes. The exact 95% confidence intervals (CIs) for binomial proportions (FN incidence and BSI proportion) were computed. Differences in binomial proportions across various cancer types were analyzed using Fisher’s exact test. In addition, the incidence of FN and the proportion of BSIs were assessed per patient. The pathogens isolated from blood cultures were also analyzed. A two-sided p value of <0.05 was considered significant. All analyses were performed using JMP Pro 16.2.0 and SAS 9.4 (SAS Institute Inc., Cary, NC, USA).

## Results

### Patient characteristics

Figure S1 depicts the patient selection process. We initially identified 157 pediatric patients as eligible. Of these, three patients with Langerhans cell histiocytosis and two receiving outpatient chemotherapy were excluded. Consequently, this analysis included 152 patients with either AML (n = 21 in total: *de novo* AML, n = 16; myeloid leukemia associated with Down syndrome [ML-DS], n = 5), ALL (n = 52 in total: B-cell acute lymphoblastic leukemia, n = 48; T-cell acute lymphoblastic leukemia, n = 4), lymphoma (n = 10 in total: Hodgkin lymphoma, n = 2; anaplastic large cell lymphoma, n = 2; Burkitt lymphoma, n = 1; diffuse large B-cell lymphoma, n = 2; extranodal natural killer/T-cell lymphoma, n = 1; lymphoblastic lymphoma, n = 2), brain tumors (n = 26 in total: intracranial germ cell tumor, n = 16; intracranial embryonal tumor, n = 8; choroid plexus tumor, n = 1; other specified intracranial neoplasm, n = 1), or solid tumors (n = 43 in total: malignant bone tumor, n = 14; soft tissue sarcoma, n = 8; malignant extracranial germ cell tumor, n = 8; neuroblastoma, n = 7; hepatic tumor, n = 3; renal tumor, n = 3). Table S1 provides the corresponding detailed diagnoses and treatment regimens.

Table 1 summarizes the patients’ baseline characteristics stratified by cancer type. The median age (range) was 2 years (0-15) for AML, 4 years (0-17) for ALL, 11 years (7-15) for lymphoma, 11 years (1-17) for brain tumors, and 8 years (0-17) for solid tumors. The median number of treatment courses (range) across all patients was 5 (1-20). Nearly all patients (n = 146, 96%) had a central venous catheter (CVC) or a peripherally inserted central catheter. None of the patients received prophylactic antibiotics during neutropenic episodes. However, all patients received sulfamethoxazole-trimethoprim prophylaxis for *Pneumocystis* pneumonia. During first-line treatment, one patient with a solid tumor succumbed to disease progression.

**Table 1.** Characteristics of Patients with Pediatric Cancer (n = 152).

	Full cohort (n = 152)	AML (n = 21)	ALL (n = 52)	Lymphoma (n = 10)	Brain tumors (n = 26)	Solid tumors (n = 43)
Age, years, median (range)	7 (0-17)	2 (0-15)	4 (0-17)	11 (7-15)	11 (1-17)	8 (0-17)
Male, n (%)	85 (56)	7 (33)	28 (54)	7 (70)	21 (81)	22 (51)
Treatment courses^a^, median (range)	5 (1-20)	5 (1-6)	5 (1-10)	5 (1-8)	5 (2-7)	6 (1-20)
Central venous catheter^b^, n (%)	146 (96)	21 (100)	50 (96)	9 (90)	24 (92)	42 (98)
Prophylactic antibiotics, n (%)	0 (0)	0 (0)	0 (0)	0 (0)	0 (0)	0 (0)
Mortality during first-line treatment, n (%)	1 (1)	0 (0)	0 (0)	0 (0)	0 (0)	1 (2)

^a^For 11 patients, some chemotherapy information could not be collected because they fell outside the range of the investigation period. ^b^The number of patients with a central venous catheter or a peripherally inserted central catheter. ALL: acute lymphoblastic leukemia; AML: acute myeloid leukemia.

### Initiation date and duration of neutropenia

Table S2 summarizes the initiation date and duration of neutropenia (neutrophil counts <500/μL and <200/μL) across different cancer types. In the AML group, neutrophil counts fell below both thresholds in all courses. This group exhibited the earliest onset of neutropenia (median, day 8) and the longest duration among all groups (median 23 and 21 days for neutrophil counts <500/μL and <200/μL, respectively).

### Proportion of BSI episodes

**Figure 1.**
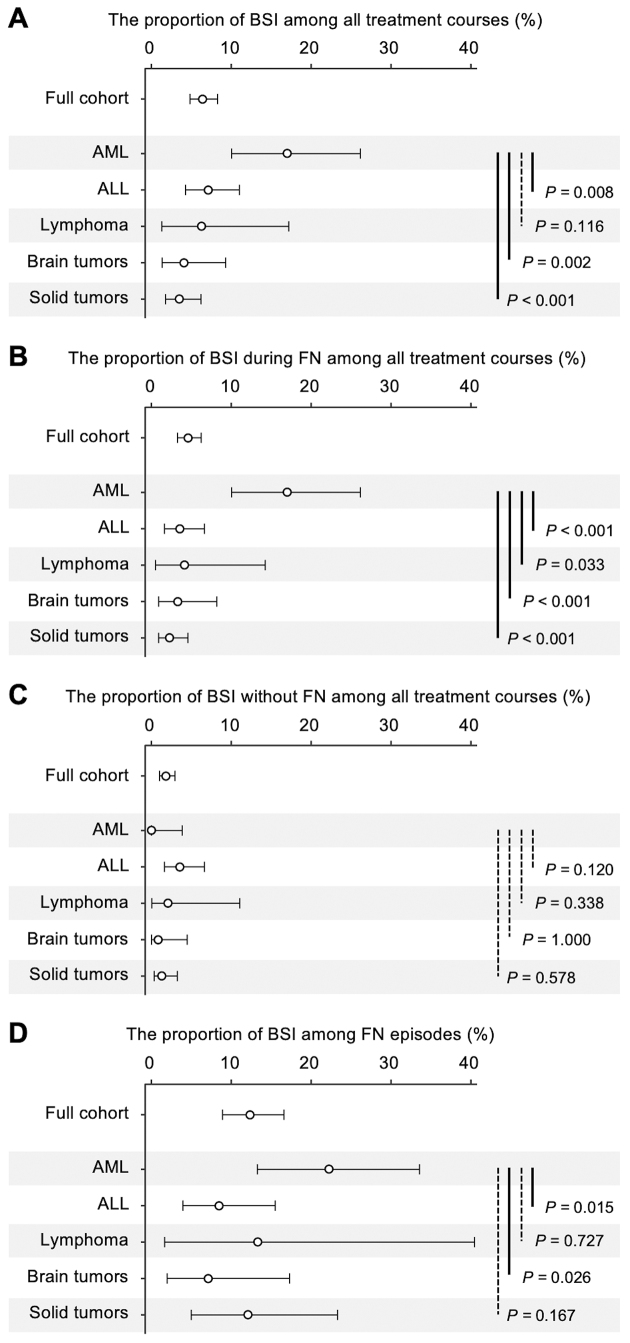
Proportion of BSI episodes in pediatric patients with cancer stratified by cancer type. (A) Proportion of BSIs across all chemotherapy courses. (B) Proportion of BSIs during FN episodes across all treatment courses. (C) Proportion of BSIs without concurrent FN across all treatment courses. (D) Proportion of BSIs among FN episodes. Point estimates with 95% confidence intervals are presented. Fisher’s exact test was used for group comparisons, with significant differences denoted by solid lines and non-significant differences by dashed lines. ALL: acute lymphoblastic leukemia; AML: acute myeloid leukemia; BT: brain tumors; BSI: bloodstream infection; FN: febrile neutropenia; ST: solid tumor.

Figure 1 depicts the incidence of BSIs in four contexts: overall BSI incidence across all treatment courses, BSIs during FN episodes across all treatment courses, BSIs without FN across all treatment courses, and BSIs among FN episodes (descriptive statistics are provided in Table 2). The AML group had the highest BSI incidence across all treatment courses (17% [95% CI: 10%-26%]), while the incidence in other groups ranged from 4% to 7% (Figure 1A). The AML group also exhibited the highest proportion of FN episodes among all treatment courses (81% [71%-88%]; Table 2). When stratified by FN status, the AML group had a significantly higher proportion of BSIs during FN among all treatment courses (17% [10%-26%]) than the ALL, lymphoma, brain tumor, and solid tumor groups (p < 0.001, p = 0.033, p < 0.001, p < 0.001, respectively; Figure 1B), with no BSIs observed in the absence of FN (Figure 1C).

**Table 2.** Proportion of FN and BSIs among Treatment Courses.

	Full cohort (829 courses)	AML (94 courses)	ALL (253 courses)	Lymphoma (48 courses)	Brain tumors (122 courses)	Solid tumors (312 courses)
FN incidence						
Courses with FN, count	326	76	113	15	62	60
Proportion of FN among all treatment courses, % [95% CI]	39% [36%-43%]	81% [71%-88%]	45% [38%-51%]	31% [19%-46%]	51% [42%-60%]	19% [15%-24%]
Blood culture test performed, count (% blood culture test performed among courses with FN)	307 (94)	72 (95)	106 (94)	15 (100)	56 (90)	58 (97)
BSI incidence						
Courses with BSI, count	53	16	18	3	5	11
Proportion of BSIs among all treatment courses, % [95% CI]	6% [5%-8%]	17% [10%-26%]	7% [4%-11%]	6% [1%-17%]	4% [1%-9%]	4% [2%-6%]
BSI during FN						
BSI during FN, count	38	16	9	2	4	7
Proportion of BSIs during FN among all treatment courses, % [95% CI]	5% [3%-6%]	17% [10%-26%]	4% [2%-7%]	4% [1%-14%]	3% [1%-8%]	2% [1%-5%]
Proportion of BSIs among FN episodes^a^, % [95% CI]	12% [9%-17%]	22% [13%-34%]	8% [4%-16%]	13% [2%-40%]	7% [2%-17%]	12% [5%-23%]
BSI without FN						
BSI without FN, count	15	0	9	1	1	4
Proportion of BSIs without FN among all treatment courses, % [95% CI]	2% [1%-3%]	0% [0%-4%]	4% [2%-7%]	2% [0%-11%]	1% [0%-4%]	1% [0%-3%]

^a^The proportion was calculated by dividing the number of BSIs during FN by the number of chemotherapy courses in which FN occurred and blood culture tests were performed. ALL: acute lymphoblastic leukemia; AML: acute myeloid leukemia; BSI: bloodstream infection; CI: confidence interval; FN: febrile neutropenia.

To investigate whether the higher incidence of BSIs in the AML group could be explained solely by the higher incidence of FN, the proportion of BSIs among FN episodes was assessed. The AML group exhibited the highest proportion of BSIs among FN episodes (22% [13%-34%]), significantly exceeding that in the ALL (22% vs. 8%, p = 0.015) and brain tumor groups (22% vs. 7%, p = 0.026; Figure 1D).

Table 3 presents the incidence of BSIs per patient. At least one BSI episode was observed in 52% of patients with AML (n = 11), 29% of patients with ALL (n = 15), 30% of patients with lymphoma (n = 3), 19% of patients with brain tumors (n = 5), and 21% of patients with solid tumors (n = 9).

**Table 3.** Number of BSI Episodes per Patient.

	Full cohort (n = 152)	AML (n = 21)	ALL^a^ (n = 52)	Lymphoma (n = 10)	Brain tumors (n = 26)	Solid tumors (n = 43)
					
Experiencing BSI(s), n (%)	43 (28)	11 (52)	15 (29)	3 (30)	5 (19)	9 (21)
Experiencing BSI(s) during FN, n (%)	32 (21)	11 (52)	9 (17)	2 (20)	4 (15)	6 (14)
Experiencing BSI(s) without FN, n (%)	13 (9)	0 (0)	8 (15)	1 (10)	1 (4)	3 (7)

^a^The total number of patients experiencing BSI(s) in ALL did not equal the sum of the breakdown because two patients experienced one episode of BSI during FN and one episode of BSI without FN. ALL: acute lymphoblastic leukemia; AML: acute myeloid leukemia; BSI: bloodstream infection; FN: febrile neutropenia.

Table S3 summarizes the occurrence dates of FN and BSI. BSIs occurred approximately 2 weeks after the treatment course initiation in the AML group (median, day 12), with similar timing observed in the lymphoma, brain tumor, and solid tumor groups (median, days 13, 15, and 14, respectively). In contrast, BSI occurred approximately 1 month after treatment course initiation in the ALL group (median, day 33).

### Pathogen profile

Gram-positive bacteria were predominantly isolated, accounting for 50 out of 53 BSI episodes (Table S4). Among patients with AML, *Streptococcus mitis* group pathogens were the most frequently isolated. Gram-negative bacteria were identified in only three cases, one in the AML group, one in the lymphoma group, and one in the solid tumor group.

### Association between BSI incidence and high-dose cytarabine in the AML and ALL groups

**Figure 2.**
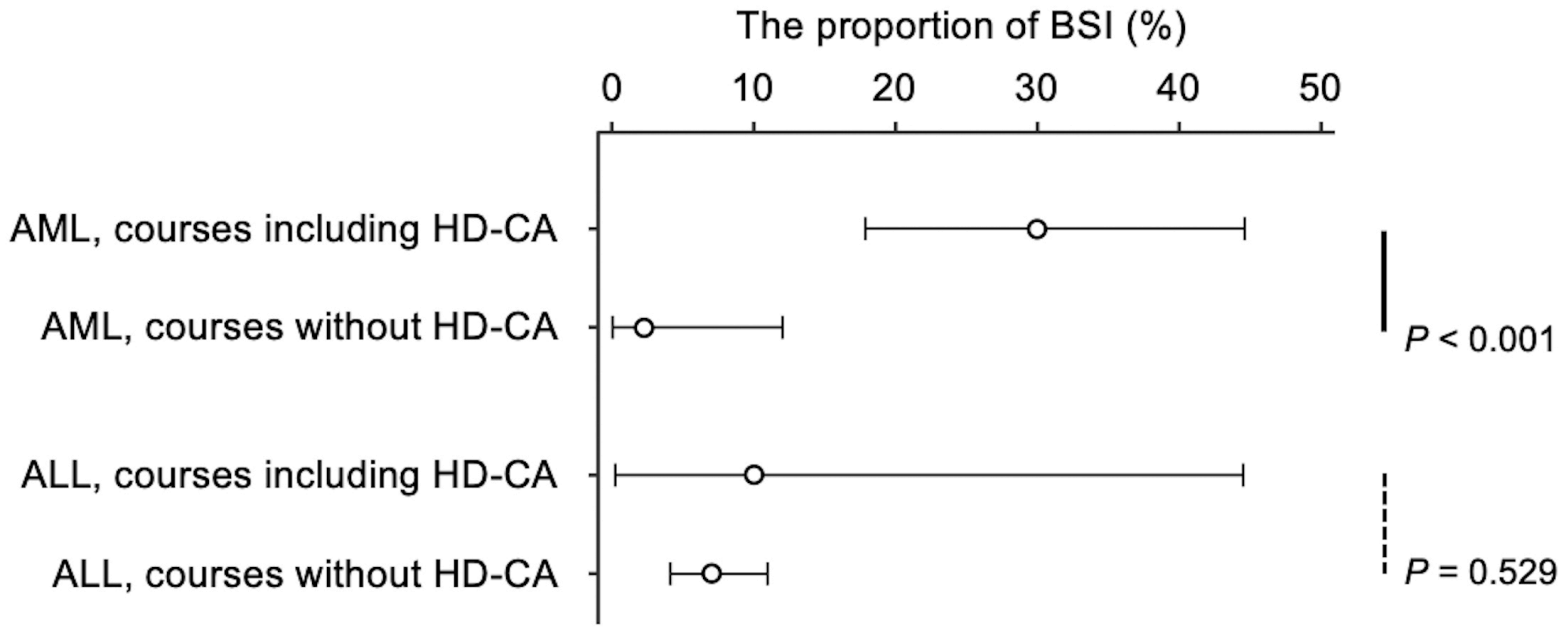
Comparative analysis of BSI incidence and proportions in pediatric patients with AML and ALL undergoing chemotherapy with and without HD-CA. BSI incidence proportions and 95% confidence intervals are displayed. Group comparisons were performed using Fisher’s exact test, with significant differences indicated by solid lines and non-significant differences by dashed lines. ALL: acute lymphoblastic leukemia; AML: acute myeloid leukemia; BSI: bloodstream infection; HD-CA: high-dose cytarabine.

High-dose cytarabine (HD-CA) use was significantly associated with an increased BSI incidence in the AML group but not in the ALL group (Figure 2; descriptive statistics in Table 4). The AML group had a markedly higher proportion of BSIs in treatment courses including HD-CA (30%), compared to those without HD-CA (2%) (p < 0.001, Fisher’s exact test), with a risk difference of 28% (95% CI: 14%-41%) and a risk ratio of 13.2 (95% CI: 2.3-365.3). Even when patients with ML-DS, who did not receive HD-CA, were excluded, the proportion of BSIs in treatments including HD-CA and without HD-CA was 30% and 5%, respectively (p = 0.052), with a risk difference of 25% (95% CI: −1% to 41%) and a risk ratio of 5.7 (95% CI: 1.0-157.8). In contrast, in the ALL group, the BSI proportion in treatment courses with and without HD-CA was 10% and 7%, respectively, with no significant association (p = 0.529). The risk difference in the ALL group was 3% (−8% to 41%) with a risk ratio of 1.4 (0.1-6.9). *Streptococcus mitis* group pathogens were predominantly isolated among patients with AML receiving HD-CA, accounting for 80% of BSIs (Table S5). The onset of neutropenia with neutrophil counts below 200/μL was significantly earlier in the treatments including HD-CA than in those without HD-CA in both the AML and ALL groups (Table S6).

**Table 4.** Relationship between BSIs and HD-CA Use in AML and ALL.

Treatment	Total courses, count	BSI, count	Proportion of BSIs, % [95% CI]	Risk difference, % [95% CI]	Risk ratio, [95% CI]
AML					
Treatment including HD-CA	50	15	30% [18%-45%]	28% [13%-43%]	13.2 [2.3-365.3]
Treatment without HD-CA	44	1	2% [0%-12%]	Reference	Reference
ALL					
Treatment including HD-CA	10	1	10% [0%-45%]	3% [−8% to 41%]	1.4 [0.1-6.9]
Treatment without HD-CA	243	17	7% [4%-11%]	Reference	Reference

ALL: acute lymphoblastic leukemia; AML: acute myeloid leukemia; BSI: bloodstream infection; CI: confidence interval; HD-CA: high-dose cytarabine.

### Association between BSI incidence and diagnostic subgroups

The incidence of BSIs was further analyzed across diagnostic subgroups. Within the AML group, *de novo* AML had a significantly higher incidence of BSIs than ML-DS (p = 0.005). No significant differences were observed in other subgroup comparisons (Table S7).

## Discussion

This study evaluated the incidence of BSIs in pediatric patients with cancer, focusing on infections occurring during FN across different cancer types. Our findings indicate that the highest incidence of BSIs was observed in patients with AML, which can be primarily attributed to the intensive chemotherapy regimens used for AML treatment. Across the different cancer groups, BSI incidence varied between 4% and 17%, with the AML cohort exhibiting a markedly higher frequency of FN (76/94, 81%) and BSI episodes (16/72, 17%) than other cancers, where BSI rates ranged from 4% to 7%. Even within hematologic malignancies, the incidence of BSIs in patients with ALL was significantly lower than that in those with AML. This disparity underscores the heightened infection risk associated with aggressive regimens that induce recurrent cycles of profound and prolonged neutropenia in AML ^(10)^. As anticipated, neutropenia in the AML group developed earlier and lasted longer than in other groups in our cohort. HD-CA is a primary therapeutic agent for AML, whereas in ALL, it is used only for high-risk patients, resulting in lower overall administration of HD-CA ^(11), (12)^. This difference in HD-CA dosing may also contribute to the lower incidence of BSIs in ALL.

In addition, our study identified a significant association between HD-CA and increased BSI incidence in the AML group. HD-CA is a crucial component of post-remission chemotherapy and is widely utilized due to its favorable impact on survival outcomes ^(13)^. Its efficacy has been validated in multiple clinical trials involving pediatric patients with AML ^(14)^, although the JPLSG AML-12 trial did not demonstrate the efficacy of adding HD-CA to the initial induction therapy ^(15)^. In our cohort, BSI incidence in AML treatment courses without HD-CA was low (2%), rising slightly to 5% after excluding patients with ML-DS. In contrast, BSI incidence in HD-CA-containing regimens was markedly higher (30%).

The increased infection risk in patients with AML receiving HD-CA is likely attributable to Viridans group streptococci (VGS). VGS commonly colonize the oral mucosa and are an important cause of sepsis in neutropenic patients with AML ^(16)^. Intravenous cefepime or vancomycin has demonstrated efficacy for VGS prophylaxis in pediatric patients with AML ^(17)^; however, current pediatric oncology guidelines do not specifically recommend prophylactic antibiotics during HD-CA treatment ^(18)^. Given the high risk of VGS infections in this setting, using prophylactic antibiotics or alternative therapeutic strategies warrants careful consideration.

Regarding pathogen profiles, gram-positive bacteria were predominant in cases of BSI. Historically, gram-negative bacteremia was more common until the late 1970s; however, a shift toward gram-positive pathogens has been observed, likely due to the widespread use of CVCs, fluoroquinolone prophylaxis, and chemotherapy-induced mucositis ^(2)^. Consistent with this trend, most BSIs in our study were caused by gram-positive bacteria, with nearly all patients having CVCs in place.

A key limitation of our study is the lack of consistent blood culture collection from all CVC lumens and peripheral sites, as recommended by the Infectious Diseases Society of America guidelines ^(19)^. This limitation was primarily due to difficulties in obtaining peripheral blood cultures in pediatric patients. Additionally, the single-center design may limit the generalizability of our findings to other institutions. Furthermore, although VGS is strongly associated with oral mucositis, we were unable to examine the incidence of oral mucositis.

In conclusion, our study delineates the epidemiology of BSIs in pediatric patients with cancer, highlighting a significantly higher incidence of BSIs in AML than in ALL, lymphoma, solid tumors, and brain tumors. In addition, HD-CA use in AML treatment was associated with a substantial increase in BSI incidence. These findings emphasize the need for further research to elucidate the pathogenesis of BSIs and explore strategies for reducing infection risk in pediatric patients with AML receiving HD-CA therapy.

## Article Information

### Conflicts of Interest

None

### Acknowledgement

The authors thank Megumi Kishita (laboratory technician) for providing the blood culture result data.

### Author Contributions

Yukihiro Matsukawa is the principal investigator and takes the main responsibility for the paper. Yukihiro Matsukawa, Kenichi Sakamoto, Kohei Takashima, Yuhachi Ikeda, Jun Matsubayashi, Makoto Osawa, Takashi Taga, and Yoshihiro Maruo designed the study. Yukihiro Matsukawa, Kenichi Sakamoto, and Jun Matsubayashi wrote the manuscript, analyzed the results, and performed the statistical analysis. All authors have discussed the results and critically reviewed the manuscript.

### Approval by Institutional Review Board (IRB)

Approval No. R2023-074, the Institutional Ethics Committee of Shiga University of Medical Science.

## Supplement

Supplementary Materials
