# Supplementary material for: Incidence of Bloodstream Infections in Pediatric Patients with Cancer during Febrile Neutropenia: A Retrospective Study
Source: JMA J. 2025 Mar 28;8(2):560–7. doi: 10.31662/jmaj.2024-0369 (PMC12095455; doi:10.31662/jmaj.2024-0369)
Supplement: Supplementary Materials [file 2433-3298-8-2-0560-s001.pdf]

Supporting Information

Incidence of Bloodstream Infections in Pediatric Patients with Cancer During Febrile Neutropenia: A Retrospective Study

Table of Contents

Figure S1: Flow chart of patient selection process 2

Table S1: Diagnostic groups and treatment protocols. 3

Table S2: The initiation date and duration of neutropenia (neutrophil count <500 /μL or 200 /μL). 6

Table S3: The date of FN and BSI occurrence..................... 7

Table S4: Frequency by type of bacteremia (gram-positive and gram-negative bacteremia). ... 8

Table S5: Pathogens isolated from blood cultures during HD-CA and non-HD-CA treatments in patients with AML. 9

Table S6: Relationship between HD-CA and the initiation date of neutropenia 10

Table S7: Relationship between BSI and diagnostic subgroups. 12

Figure S1: Flow chart of patient selection process

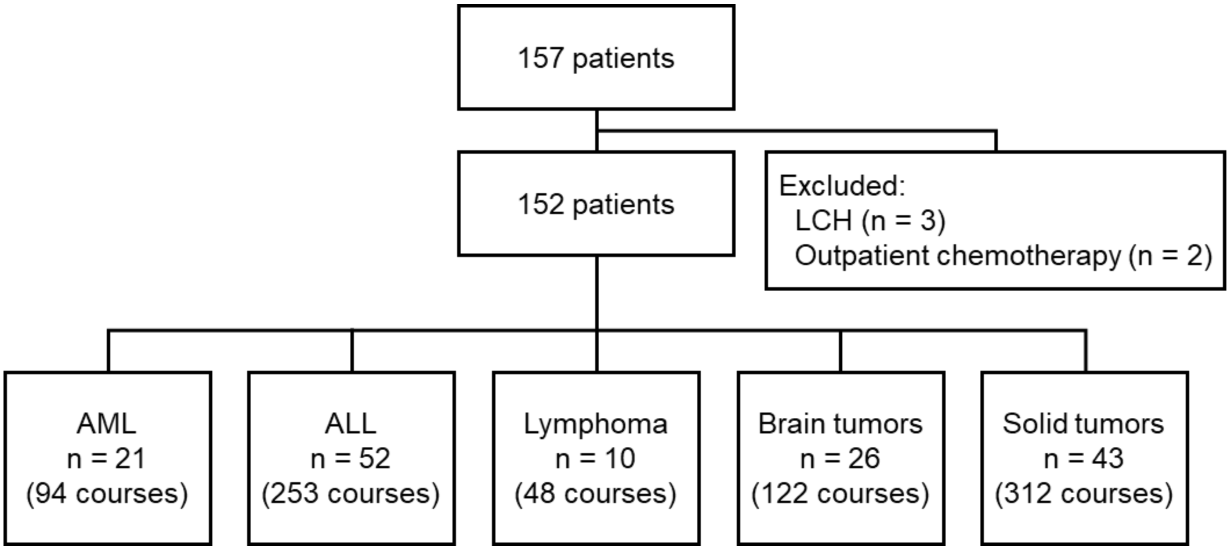

Table S1: Diagnostic groups and treatment protocols.

Group	Subgroup: ***n***	Protocol or treatment	***n***
AML	*de novo* AML: 16<br>ML-DS: 5	JPLSG-AML05	7
		JPLSG-AML-12	5
		JPLSG-AML-20	4
		JPLSG-AML-D11	2
		JPLSG-AML-D16	3
ALL	B-ALL: 48<br>T-ALL: 4	CCLSG-ALL2004	6
		JPLSG-MLL-10	1
		JPLSG-ALL-B12	35
		JPLSG-ALL-B19	6
		JPLSG-ALL-T11	3
		JPLSG-ALL-T19	1
Lymphoma	Hodgkin lymphoma: 2<br>ALCL: 2<br>Burkit Lymphoma: 1<br>DLBCL: 2<br>ENKL: 1<br>LBL: 2	CCLSG-NHL960	1
		JPLSG-ALB-NHL03	1
		JPLSG-B-NHL03	2
		JPLSG-B-NHL14	2
		JPLSHG-HL14	2
		RT-2/3DeVIC+SMILE+ESCAP treatment	1
		FRE-IGR ALCL99	1

Table S1 (continued.)

Brain tumor	Intracranial germ cell tumor: 16<br>Intracranial embryonal tumor: 8<br>Choroid plexus tumor: 1<br>Other specified intracranial neoplasms: 1	JPBTC-medulloblastoma regimen	8
		JPBTC-infant medulloblastoma regimen	1
		JPBTC-germinoma regimen	16
		JPLSG-AT20	1
Solid tumor	Malignant bone tumor:14<br>Soft tissue sarcoma: 8<br>Malignant extracranial germ cell tumor: 8<br>Neuroblastoma: 7<br>Hepatic tumor: 3<br>Renal tumor: 3	NECO95-J	7
		Osteosarcoma-details unknown	3
		Rhabdomyosarcoma-VAC-based regimen	8
		JNBSG LI-D regimen	3
		JNBSG JN-H-15	2
		Neuroblastoma-James treatment	1
		VDC/IE chemotherapy	5
		BEP chemotherapy	3
		JEB chemotherapy	5
		JWiTS-2 protocol	3
		JPLT: Pediatric Hepatic International Tumor treatment	3

Abbreviations: ALCL, anaplastic large cell lymphoma; ALL, acute lymphoblastic leukemia; AML, acute myeloid leukemia; B-ALL, B-cell acute lymphoblastic leukemia; BEP, bleomycin, etoposide, and platinum; CCLSG, Children's Cancer & Leukemia Study Group; DLBCL, diffuse large B-cell lymphoma; ENKL, extranodal NK/T-cell lymphoma; JEB, carboplatin, etoposide,

and bleomycin; JNBSG, Japan Neuroblastoma Study Group; JPBTC, Japanese Pediatric Brain Tumor; JPLSG, Japan Pediatric Leukemia/Lymphoma Study Group; JPLT, Japanese Study Group for Pediatric Liver Tumor; JWiTS, Japan Wilms Tumor Study Group; LI-D, vincristine, cyclophosphamide, pirarubicin, and cisplatin; LBL, lymphoblastic lymphoma; ML-DS, myeloid leukemia associated with Down syndrome; NECO, neoadjuvant chemotherapy for osteosarcoma; T-ALL, T-cell acute lymphoblastic leukemia; VAC, vincristine, dactinomycin, and cyclophosphamide; VDC/IE, vincristine, doxorubicin, cyclophosphamide alternating with ifosfamide, etoposide.

Table S2: The initiation date and duration of neutropenia (neutrophil count <500 /µL or <200 /µL).

	AML	ALL	Lymphoma	Brain tumors	Solid tumors
Number of courses examined[a], count	17	64	18	22	56
Neutrophil count <500 /µL					
Number of courses, count (%)	17 (100%)	53 (83%)	10 (56%)	21 (95%)	33 (59%)
Initiation date, median (range)	8 (1, 15)	13 (1, 32)	14 (10, 17)	11 (8, 16)	12 (3, 24)
Duration, day, median (range)	23 (13, 122)	22 (2, 50)	10 (2, 21)	9 (4, 13)	10 (2, 22)
Neutrophil count <200 /µL					
Number of courses, count (%)	17 (100%)	49 (77%)	9 (50%)	20 (91%)	20 (36%)
Initiation date, median (range)	8 (1, 15)	17 (1, 39)	16 (10, 19)	12 (8, 18)	14 (10, 25)
Duration, day, median (range)	21 (13, 115)	14 (2, 35)	5 (3, 16)	7 (4, 13)	6 (1, 15)

[a] Only the chemotherapy courses administered during 2020–2023 were examined.

Abbreviations: ALL, acute lymphoblastic leukemia; AML, acute myeloid leukemia.

Table S3: FN and BSI occurrence dates.

	AML	ALL	Lymphoma	Brain tumors	Solid tumors
FN occurrence date, median (range)	14 (8, 30)	28 (4, 61)	15 (6, 37)	14 (9, 22)	13 (6, 26)
BSI occurrence date, median (range)	12 (10, 16)	33 (15, 54)	13 (5, 19)	15 (12, 18)	14 (2, 26)

Abbreviations: ALL, acute lymphoblastic leukemia; AML, acute myeloid leukemia. BSI, bloodstream infection; FN, febrile neutropenia.

Table S4: Frequency by type of bacteremia (gram-positive and gram-negative bacteremia).

	**AML (16 BSI episodes)**	**ALL (18 BSI episodes)**	**Lymphoma (3 BSI episodes)**	**Brain tumors (5 BSI episodes)**	**Solid tumors (11 BSI episodes)**
Gram-positive bacteria, count (%)					
Staphylococci	1 (6%)	5 (28%)	1 (33%)	4 (80%)	5 (45%)
Streptococcus mitis group	13 (81%)	4 (22%)	1 (33%)	0 (0%)	5 (45%)
Others	1 (6%)	9 (50%)	0 (0%)	1 (20%)	0 (0%)
Gram-negative bacteria, count (%)	1 (6%)	0 (0%)	1 (33%)	0 (0%)	1 (9%)

Abbreviations: ALL, acute lymphoblastic leukemia; AML, acute myeloid leukemia; BSI, bloodstream infection.

Table S5: Pathogens isolated from blood cultures during HD-CA and non-HD-CA treatments in patients with AML.

Blood culture isolated pathogen	Treatment including HD-CA (15 courses)	Treatment without HD-CA (1 course)
Streptococcus mitis group, count (%)	12 (80%)	1 (100%)
Pseudomonas aeruginosa, count (%)	1 (7%)	0 (0%)
Bacillus species, count (%)	1 (7%)	0 (0%)
Staphylococcus epidermidis, count (%)	1 (7%)	0 (0%)

Abbreviations: AML, acute myeloid leukemia; HD-CA, high-dose cytarabine.

Table S6: Relationship between HD-CA and the initiation date of neutropenia.

	Treatment including HD-CA	Treatment without HD-CA	*P* value[a]
AML			
Number of courses examined, count	50	44	
Neutrophil count <500 /µL			
Number of courses, count (%)	50 (100)	43 (98)	0.468
Initiation date, median (range)	8 (3, 10)	9 (1, 15)	0.157
Neutrophil count <200 /µL			
Number of courses, count (%)	50 (100)	42 (95)	0.216
Initiation date, median (range)	8 (4, 11)	11 (1, 18)	0.006
ALL			
Number of courses examined[b], count	10	58	
Neutrophil count <500 /µL			
Number of courses, count (%)	10 (100)	47 (81)	0.197
Initiation date, median (range)	9 (1, 16)	15 (1, 32)	0.038
Neutrophil count <200 /µL			
Number of courses, count (%)	9 (90)	43 (74)	0.432
Initiation date, median (range)	12 (1, 18)	21 (1, 39)	0.028

[a] Mann–Whitney *U* test and Fisher's exact test for continuous and categorical variables, respectively.

[b] Only the chemotherapy courses without HD-CA administered during 2020–2023 were examined.

Abbreviations: ALL, acute lymphoblastic leukemia; AML, acute myeloid leukemia; HD-CA, high-dose cytarabine.

Table S7: Relationship between BSI and diagnostic subgroups.

Diagnosis	Total courses, count	Courses with BSI, count (%)	*P* value[a]
AML			
de novo AML	69	16 (23%)	0.005
ML-DS	25	0 (0%)	
ALL			
B-ALL	233	16 (7%)	0.642
T-ALL	20	2 (10%)	
Lymphoma			
Lymphoblastic lymphoma	8	1 (13%)	0.429
Other than lymphoblastic lymphoma	40	2 (5%)	
Brain tumor			
Medulloblastoma	30	3 (10%)	0.095
Other than medulloblastoma	92	2 (2%)	

Table S7 (continued.)

Solid tumor			
Neuroblastoma, rhabdomyosarcoma, and malignant bone tumors	170	8 (5%)	0.356
Other solid tumors[b]	142	3 (2%)	

[a] Fisher's exact test. [b] Other solid tumors include renal tumors, hepatic tumors, and malignant extracranial germ cell tumor.

Abbreviations: AML, acute myeloid leukemia; B-ALL, B-cell acute lymphoblastic leukemia; BSI, bloodstream infection; ML-DS, AML associated with Down syndrome; T-ALL, T-cell acute lymphoblastic leukemia.
